# A cost-effectiveness analysis of a novel algorithm to sequentially diagnose leprosy based on manufactured tests under the SUS perspective

**DOI:** 10.1590/0102-311XEN038723

**Published:** 2024-01-08

**Authors:** Milene Rangel da Costa, Carlos Alberto da Silva Magliano, Bruno Monteiro Barros, Quenia Cristina Dias Morais, Andressa Araujo Braga, Kátia Marie Simões e Senna, Ciro Martins Gomes, Alexandre Casimiro de Macedo, Marisa da Silva Santos

**Affiliations:** 1 Instituto Nacional de Cardiologia, Rio de Janeiro, Brasil.; 2 Universidade Federal do Rio de Janeiro, Rio de Janeiro, Brasil.; 3 Instituto Nacional de Traumatologia e Ortopedia Jamil Haddad, Rio de Janeiro, Brasil.; 4 Universidade de Brasília, Brasília, Brasil.; 5 Secretaria de Vigilância em Saúde e Ambiente, Ministério da Saúde, Brasília, Brasil.

**Keywords:** Leprosy, Routine Diagnostic Test, Cost Effectiveness, Hanseníase, Testes Diagnósticos de Rotina, Custo-Efetividade, Lepra, Pruebas Diagnósticas de Rutina, Costo Efectividad

## Abstract

Brazil has the second largest number of leprosy cases (a disease with a significant burden) in the world. Despite global and local efforts to eliminate this public health problem, inadequate or late diagnosis contribute to perpetuate its transmission, especially among household contacts. Tests such as the rapid IgM antibody detection (RT) and real-time polymerase chain reaction (RT-PCR) were developed to overcome the challenges of early diagnosis of leprosy. This study aimed to analyze the cost-effectiveness of a new diagnostic algorithm recommended by the Brazilian government to diagnose leprosy in household contacts of confirmed leprosy cases, which includes the RT and RT-PCR tests. A decision tree model was constructed and the perspective of the Brazilian Unified National Health System (SUS) and a 1-year time horizon were adopted. Only direct medical costs related to diagnostic tests were included. Effectiveness was measured as the number of avoided undiagnosed leprosy cases. Different scenarios were analyzed. The sequential use of RT, slit-skin smear (SSS) microscopy, and RT-PCR as recommended by the Brazilian Ministry of Health was compared to a base case (isolated SSS microscopy), yielding an incremental cost-effectiveness ratio of USD 616.46 per avoided undiagnosed leprosy case. Univariate sensitivity analysis showed that the prevalence of leprosy among household contacts was the variable that influenced the model the most. This is the first economic model to analyze a diagnostic algorithm of leprosy. Results may aid managers to define policies and strategies to eradicate leprosy in Brazil.

## Resumo

O Brasil tem o segundo maior número de casos de hanseníase (doença com carga significativa) do mundo. Apesar dos esforços globais e locais para eliminar esse problema de saúde pública, o diagnóstico inadequado ou tardio contribui para perpetuar sua transmissão, especialmente entre contatos intradomiciliares. Exames como o teste rápido de anticorpos IgM (RT) e a reação em cadeia da polimerase em tempo real (RT-PCR) foram desenvolvidos para superar as barreiras do diagnóstico precoce da hanseníase. Este estudo teve como objetivo analisar a relação custo-efetividade de um novo algoritmo de diagnóstico recomendado pelo governo brasileiro para diagnosticar a hanseníase em contatos domiciliares de casos confirmados de hanseníase, que inclui os testes RT e RT-PCR. Foi construído um modelo de árvore de decisão e adotada a perspectiva do Sistema Único de Saúde (SUS) considerando o período de um ano. Foram incluídos apenas os custos médicos diretos relacionados aos exames diagnósticos. A efetividade foi medida considerando o número de casos evitados de hanseníase. Diferentes cenários foram analisados. O uso sequencial de RT, baciloscopia e RT-PCR, conforme recomendado pelo Ministério da Saúde, foi comparado a um caso base (baciloscopia isolada), obtendo-se uma razão de custo-efetividade incremental de USD 616,46 por caso evitado de hanseníase. A análise de sensibilidade univariada mostrou que a prevalência de hanseníase entre contatos intradomiciliares foi a variável que mais influenciou o modelo. Este é o primeiro modelo econômico a analisar um algoritmo diagnóstico da hanseníase. Os resultados poderão auxiliar os gestores na definição de políticas e estratégias para a erradicação da hanseníase no Brasil.

## Resumen

Brasil tiene el segundo mayor número de casos de lepra (enfermedad con carga significativa) del mundo. A pesar de los esfuerzos globales y locales para eliminar ese problema de salud pública, el diagnóstico inadecuado o tardío contribuye a perpetuar su transmisión, sobre todo entre contactos intradomiciliarios. Los exámenes como la prueba rápida de anticuerpos IgM (RT) y la reacción en cadena de la polimerasa en tiempo real (RT-PCR) se desarrollaron para superar las barreras del diagnóstico precoz de la lepra. El objetivo de este estudio fue analizar la relación de costo-efectividad de un nuevo algoritmo de diagnóstico recomendado por el gobierno brasileño para diagnosticar la lepra en contactos domiciliarios de casos confirmados de lepra, que incluye las pruebas RT y RT-PCR. Se construyó un modelo de árbol de decisión y se adoptó la perspectiva del Sistema Único de Salud (SUS) teniendo en cuenta el periodo de un año. Solo se incluyeron los costos médicos directos relacionados con los exámenes diagnósticos. Se midió la efectividad teniendo en cuenta el número de casos de lepra evitados. Se analizaron distintos escenarios. Se comparó el uso secuencial de RT, baciloscopia y RT-PCR, conforme el Ministerio de Salud recomienda, con un caso base (baciloscopia aislada), y se obtuvo un cociente de costo-efectividad incremental de USD 616,46 por cada caso de lepra evitado. El análisis de sensibilidad univariante mostró que la prevalencia de lepra entre contactos intradomiciliarios fue la variable que más influyó el modelo. Este es el primer modelo económico que analiza un algoritmo diagnóstico de lepra. Los resultados podrán ayudar los gestores a definir políticas y estrategias para erradicar la lepra en Brasil.

## Introduction

Leprosy is a chronic infectious disease caused by *Mycobacterium leprae* and *Mycobacterium lepromatosis* that affects the skin and the peripheral nervous system. It is considered one of the oldest and most neglected diseases in the world, remaining endemic in several countries, especially those with prevailing precarious living conditions and difficulties of access to health services ^1^.

Despite the significant reduction in the number of cases since the availability of polychemotherapy in the 1980s, leprosy detection rate has remained stable in recent years ^2^. Data from the World Health Organization (WHO) show about 127,396 cases of leprosy worldwide in 2020 ^2^. The incidence of the disease remains high, and most emerging cases occur in countries such as Brazil, India, Nepal, China, and some African countries such as Angola and Mozambique ^3^ ^,^ ^4^.

Although the number of new cases has gradually decreased over the past years, Brazil accounts for 96.3% of all leprosy cases in the Americas and is the only American country that has failed to achieve the WHO leprosy control goal of less than one case per 10,000 inhabitants ^5^. With a detection rate of 1.32 cases per 10,000 inhabitants, Brazil has the second highest number of leprosy cases in the world (a disease with a significant burden) ^6^. According to the Brazilian Ministry of Health data, the most important leprosy case cluster in Brazil lies in Mato Grosso State, which is considered the area with the highest risk of infection. Other high risk areas include Tocantins, southern Pará, and northern Goiás ^7^.

Individuals affected by leprosy may have a broad spectrum of clinical and histopathological manifestations depending on individual patterns of immune responses to infections ^8^ ^,^ ^9^. The disease can affect sight, joints, the upper respiratory tract, and adrenal glands ^10^. According to the WHO classification system, patients can be categorized as paucibacillary (PB), if they have up to five lesions, or multibacillary (MB), if the number of skin lesions exceeds five or if patients test positive in slit-skin smear (SSS) microscopy, regardless of the number of skin lesions ^11^. MB patients have a high potential for transmissibility and a higher risk of relapse and progression to reactive episodes ^12^ ^,^ ^13^. Moreover, treatment duration increases from six (for PB) to 12 months (for MB) ^11^.

Untreated leprosy can cause progressive deformations, pain, and physical limitations, which have a devastating impact on patients’ quality of life, making early detection essential ^14^. Leprosy is an insidious disease whose symptoms usually emerge between two and six years after the infection but may take up to 20 years, during which transmission is possible. Thus, inadequate or late diagnoses contribute to perpetuating the transmission of the disease, especially among MB patients’ household contacts ^15^.

However, the accurate diagnosis and prompt treatment of leprosy patients remain a challenge. Its detection is mainly based on clinical examinations, which can be difficult for untrained physicians ^16^. Additionally, some forms of PB leprosy can be easily mistaken for other dermatological conditions, such as granuloma annulare, cutaneous sarcoidosis, or pityriasis alba. In fact, about 30% of cases (many involving MB patients in the incubation period) show no typical symptoms, such as the presence of skin areas with loss of sensitivity ^17^.

Laboratory tests can complement clinical examination, of which SSS microscopy is the most widely used test. It consists of identifying *M. leprae* in intradermal scrapings obtained from specific collection sites ^2^. However, despite its high specificity, this test has low sensitivity ^18^ and up to 70% of infected individuals show negative results ^19^. Moreover, this invasive test causes significant discomfort to patients and requires experienced professionals to collect and analyze samples.

Additional tests have been recently developed to improve the early detection of leprosy, reduce its transmission, and improve patients’ prognosis, including tests based on the detection of IgM antibodies against phenolic glycolipid-I (anti-PGL-I, a specific *M. leprae* antigen) and molecular tests using polymerase chain reaction (PCR) techniques. Both have good sensitivity for detecting MB cases but not for PB ones ^20^. Although they cannot be used alone as diagnostic tests, they can be useful in conjunction with other clinical and diagnostic data ^21^.

Given the remaining challenges and in line with the *WHO Global Strategy for Leprosy 2021-2030* ^4^, the Brazilian government has been adopting national policies to combat leprosy and reduce its burden in the country ^22^. Among its proposals, the Brazilian Ministry of Health incorporated a new diagnostic flow to evaluate suspected leprosy cases. This novel diagnostic algorithm includes serological and PCR tests in addition to clinical examinations and SSS microscopy to investigate suspected cases among infected people’s household contacts. The serological test has the advantage of being disease-specific, user-friendly, quick, and easy to perform in end users ^23^. PCR can accurately identify *M. leprae* and is greatly important as a confirmatory test ^24^. Together, these tests may potentially reduce the number of undiagnosed cases and help to avoid unnecessary SSS tests, which cause great discomfort to patients.

Brazil was the first country to make the rapid serological test to detect *M. leprae* available in its health system, a measure aimed at better controlling the disease ^6^. As leprosy control activities are decentralized throughout the country, primary care prevents, diagnoses, and treats the disease ^25^ ^,^ ^26^. Hence, healthcare providers working at primary care facilities and family health teams must be prepared to recognize the early signs of leprosy, diagnose it, and recommend proper control measures and treatment for patients ^26^. The new Brazilian Ministry of Health diagnostic flow to evaluate suspected leprosy cases aimed to improve the diagnostic capacity of primary care by providing rapid and easy-to-perform tests that dispense specialized personnel. The *Brazilian National Guidelines for Leprosy* ^6^ recommend the test to identify cases in confirmed patients’ household contacts and ensure its availability at primary care.

Given the importance of early diagnosis to interrupt transmission (especially among high-risk individuals such as patients’ household contacts), this diagnostic strategy is fundamental to achieve the goals established by the WHO toward zero leprosy, i.e., no infections, cases, disabilities, and related stigma ^4^.

This study aimed to analyze the costs and consequences of the aforementioned new algorithm for the diagnosis of leprosy that has been incorporated into the Brazilian Unified National Health System (SUS). Economic evaluations are useful to better evaluate the resource implications of decisions and to generate vital information on the obtained benefits. Here, we describe a cost-effectiveness analysis that compared the new algorithm to diagnose leprosy in intradomiciliary contacts of patients with leprosy to the previous recommended procedures to diagnose the disease in this population under the SUS perspective.

## Materials and methods

This is a cost-effectiveness analysis from the payer’s perspective, i.e., SUS, which is considered relevant to prevent and monitor leprosy in Brazil, as well as treat patients within the SUS. A 1-year time horizon was chosen to conservatively estimate the short-term clinical and economic impacts of the intervention. Given this short time horizon, no discount rate was applied.

The model considered a hypothetical cohort of 100 individuals. The base case population consisted of intradomiciliary contacts of leprosy cases who were aged 18 years or above and showed suspected dermatological or neurological lesions. This is considered a high-risk population and, according to specialists, the prevalence of leprosy in this group revolves around 15%. No subgroups were analyzed. The tests are considered to be undertaken at primary care in any location in Brazil.

**Figure 1:**
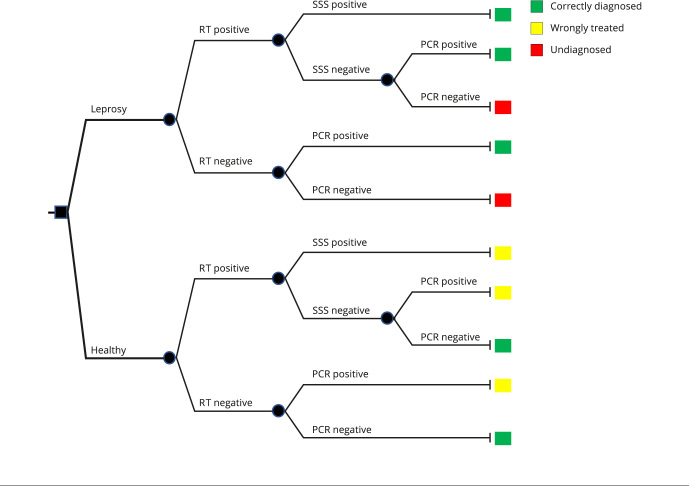
Decision tree representing the new diagnostic algorithm proposed by the Brazilian Ministry of Health.

PCR: polymerase chain reaction; SSS: slit-skin smear; RT: immunochromatographic rapid test.

A decision tree model was developed using Microsoft Excel (https://products.office.com/) to calculate the costs and benefits of the comparators. Its structure represented the comparison of diagnostic flowcharts as in Figure 1 and Supplementary Material (Figure S1; https://cadernos.ensp.fiocruz.br/static//arquivo/suppl-e00038723_8950.pdf). According to the new diagnostic algorithm proposed by the Health Surveillance Secretariat of the Brazilian Ministry of Health (SVS/MS), previously exposed individuals with suspicious lesions are subjected to rapid immunochromatographic testing (RT) to qualitatively determine anti-*M. leprae* IgM antibodies. If reactive, individuals are subjected to SSS microscopy. A positive result in the latter confirms the diagnosis of leprosy. However, in case of a negative result, a PCR test is performed. If RT finds a nonreactive result, individuals are subjected to a confirmatory PCR, which may confirm or reject the diagnosis. RT is an immunochromatographic flow test (ML Flow test) for the detection of antibodies to the phenolic glycolipid-I (PGL-I) of *M. leprae*. This simple and quick test with serum and whole-blood samples provides results in 10 minutes ^27^. In this analysis, the properties of the RT and PCR tests were considered analogous to the following manufactured technologies: RT to qualitatively determine anti-*M. leprae* IgM antibodies in biological samples (serum, plasma, or whole blood); BIOCLIN FAST ML-FLOW (Quibasa Química Básica Ltda., https://www.bioclin.com.br/); and real-time PCR targeting 16SrRNA, RLEP, and 18SrRNA, Kit NAT HANSENÍASE (Institute of Molecular Biology of Paraná; https://www.ibmp.org.br/).

As a comparator, the previous diagnostic flow, in which only SSS microscopy is performed (Supplementary Material - Figure S1; https://cadernos.ensp.fiocruz.br/static//arquivo/suppl-e00038723_8950.pdf), was considered. This diagnostic approach has several limitations. SSS microscopy has low sensitivity for both PB and MB cases (25 and 62%, respectively) ^18^. This painful procedure negatively affects suspected case adherence to testing. Also, the test must be carried out by qualified personnel. These features contribute to a significant number of false negative results and thus to disease transmission.

Additionally, two alternative scenarios were evaluated. The intervention was compared to other diagnostic approaches: (1) the use of a RT followed by SSS microscopy (without PCR) (Supplementary Material - Figure S2; https://cadernos.ensp.fiocruz.br/static//arquivo/suppl-e00038723_8950.pdf) and (2) the use of PCR by itself (Supplementary Material - Figure S3; https://cadernos.ensp.fiocruz.br/static//arquivo/suppl-e00038723_8950.pdf). Scenario analysis aimed to explore different possibilities of incorporating the RT and PCR tests to diagnose leprosy and assess their cost-effectiveness. These scenarios were proposed by experts and the model is available upon request.

### Model parameters

#### Probabilities

The sensitivity and specificity of the diagnostic tests were obtained by a quick review of the literature followed by the estimation of a meta-analysis using the bivariate hierarchical model ^28^ on R software, version 4.1 (http://www.r-project.org). The methodology of the rapid review is shown in the Supplementary Material (https://cadernos.ensp.fiocruz.br/static//arquivo/suppl-e00038723_8950.pdf), including the search strategies for PCR and RT (Tables S1 and S2), *Preferred Reporting Items for Systematic Reviews and Meta-Analyses* (PRISMA) diagrams (Figures S4 and S5), and meta-analysis results for test sensibility and specificity (Table S3).

The prevalence of leprosy among household contacts and the percentage of MB patients in Brazil were obtained from a panel of experts ^29^, which included three experienced specialists from the Brazilian Ministry of Health (two clinicians and one pharmacist).

#### Costs

Only the direct medical costs associated with diagnosis (which included the RT, PCR, and SSS microscopy test costs) were included in this analysis. A maximum of one unit of each test per patient was considered. The cost of SSS microscopy was obtained from SIGTAP (Management System of the Table of Procedures, Medications, Orthotics, Prosthetics, and Special Materials from the SUS) ^30^ and adjusted by a 2.8 factor to compensate for the fact that the costs obtained from SIGTAP only refer to federal spending and do not adequately represent national cost variability. The 2.8 value was derived from a large study conducted by the Brazilian Ministry of Health that recommends its use in economic evaluations of SUS health technologies ^31^.

The cost of the RT (USD 4.80) was obtained by a pricing survey of the Brazilian market and that of PCR - which includes the cost of the NAT Leprosy Kit (USD 19.40), the DNA extraction kit (USD 4.80), and other supplies (USD 2.00) - was provided by the SVS/MS. All costs were converted to dollars considering the mean conversion rate of BRL 5.00 per USD according to the Central Bank of Brazil on 28th April, 2023 ^32^.

Costs related to the treatment of diagnosed cases were not included in the analysis. Polychemotherapy drugs are currently donated by the WHO under a humanitarian agreement at no cost for the Brazilian government. Regarding human resources and infrastructure, according to Brazilian Ministry of Health experts, the implementation of the new diagnostic flow would not require the expansion of facilities, equipment acquisition, and work force hiring. Moreover, it was assumed that patients and health care providers would show total adherence to the new Brazilian Ministry of Health recommendations.

The values of all parameters in the model were reviewed by a panel of experts to make the analyses more conservative and appropriate to the Brazilian reality. Parameter values are summarized in Table 1.

**Table 1:** Input parameters of the decision tree model to diagnose leprosy and analyze sensitivity.

Variables	Base case	Sensitivity analysis	Source
Leprosy prevalence among household contacts of confirmed cases (%)	15	2-20	Panel of experts
Proportion of MB patients (%)	70	50-90	Panel of experts
SSS microscopy sensitivity for PB leprosy (%)	25	0-50	Meta-analysis
SSS microscopy specificity for PB leprosy (%)	100	100-100	Meta-analysis
SSS microscopy sensitivity for MB leprosy (%)	62	30-80	Meta-analysis
SSS microscopy specificity for MB leprosy (%)	100	100-100	Meta-analysis
RT sensitivity for PB leprosy (%)	23.3	13.2-37.6	Meta-analysis
RT specificity for PB leprosy (%)	91.5	81.6-96.3	Meta-analysis
RT sensitivity for MB leprosy (%)	81.8	61.7-92.6	Meta-analysis
RT specificity for MB leprosy (%)	89.1	84.5-92.5	Meta-analysis
PCR sensitivity for PB leprosy (%)	57	41-70	Meta-analysis
PCR specificity for PB leprosy (%)	90	85-95	Meta-analysis
PCR sensitivity for MB leprosy (%)	80	70-90	Meta-analysis
PCR specificity for MB leprosy (%)	95	90-100	Meta-analysis
RT cost (USD)	4.80	3.84-5.76	Pricing survey
PCR cost (USD)	26.20	20.96-31.44	Pricing survey
SSS microscopy cost (USD)	2.35	2.35-2.35	Brazilian Ministry of Health

MB: multibacillary; PB: paucibacillary; PCR: polymerase chain reaction; SSS: slit-skin smear; RT: immunochromatographic rapid test.

### Measure of effectiveness

As no data on utilities, survival, or other important outcomes for patients in Brazil were found in the literature, the number of avoided undiagnosed leprosy cases was chosen as a measure of effectiveness. It was based on the hypothesis that the intervention should decrease false-negative diagnosis.

This outcome was considered appropriate since, according to the literature, about half of all leprosy cases are not reported, denoting identification failures ^33^. Early diagnosis is essential to reduce transmissibility and avoid the disabling lesions and stigma associated with the disease.

### Sensitivity analysis

Univariate and probabilistic sensitivity analyses were performed to test the robustness of the model. All model parameters were included in the analyses. For the probabilistic analysis, beta probability distribution was adopted for probability parameters and gamma distribution was adopted for cost parameters. A total of 1,000 simulations were performed for the probabilistic analysis.

## Results

The cost of tracking individuals with suspected lesions using only SSS microscopy (previous diagnostic practice) was USD 235.20 per 100 tested individuals. All other analyzed scenarios showed higher costs (Table 2). The use of the RT followed by SSS microscopy (alternative scenario 1) costed USD 523.02 per 100 tested contacts. Conversely, alternative scenario 2 (only PCR) costed USD 2,620.00. The new Brazilian Ministry of Health diagnostic flow, which sequentially performs the RT, SSS, and PCR tests costed USD 2,996.02 per 100 screened people. Despite its higher cost, this strategy reduced the number of undiagnosed cases (a 61% relative risk reduction) the most when compared to only SSS microcopy (7.33 vs. 2.85 undiagnosed cases per 100 patients) (Table 2).

**Table 2:** Cost-effectiveness results for different diagnostic strategies per 100 persons tested.

Strategy	Costs (USD)	Undiagnosed cases (per 100 patients)	Incremental cost (USD)	Incremental effectiveness	ICER
SSS	235.20	7.33	-	-	-
RT+SSS+PCR	2,996.02	2.85	2,760.82	4.48	616.46
Alternative scenario 1 (RT+SSS)	523.02	9.39	-	-	Dominated
Alternative scenario 2 (PCR)	2,620.00	4.04	2,384.80	3.29	723.62

ICER: incremental cost-effectiveness ratio; PCR: polymerase chain reaction; SSS: slit-skin smear; RT: immunochromatographic rapid test.

Alternative scenario 1 (RT followed by SSS microscopy) was considered dominated for its higher cost and number of undiagnosed cases than only SSS microscopy (9.39 vs. 7.33 undiagnosed cases per 100 tested patients). Conversely, the new Brazilian Ministry of Health diagnostic flow and alternative scenario 2 showed higher costs and greater effectiveness. However, the former showed a lower incremental cost-effectiveness ratio (ICER) (USD 616.46 vs. USD 723.62), configuring the most cost-effective analyzed scenario. Table 2 shows all results.

**Figure 2:**
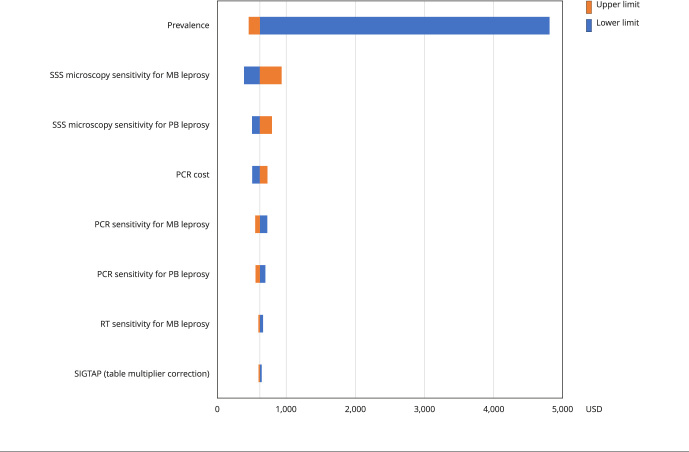
Results of the deterministic sensitivity analysis for a novel algorithm for the sequential diagnosis of leprosy based on manufactured tests.

MB: multibacillary; PB: paucibacillary; PCR: polymerase chain reaction; SIGTAP: Management System of the Table of Procedures, Medications, Orthotics, Prosthetics, and Special Materials from the SUS; SSS: slit-skin smear; RT: immunochromatographic rapid test.

**Figure 3:**
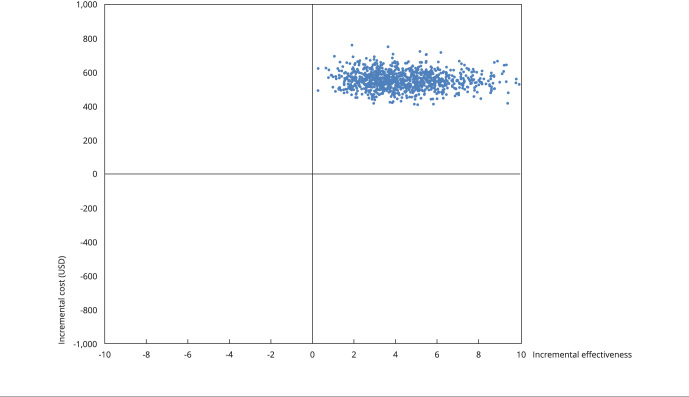
Results of probabilistic sensitivity analysis for a novel algorithm for the sequential diagnosis of leprosy based on manufactured tests.

Univariate sensitivity analysis showed that the prevalence of leprosy among contacts and the sensitivity of SSS microscopy in PB and MB patients were the most important variables for the model (Figure 2). Prevalence reduction increases ICER. The other variables failed to substantially affect the model. In the probabilistic sensitivity analysis, all simulations remained in the upper right quadrant of the cost-effectiveness plan; i.e., the new Brazilian Ministry of Health diagnostic algorithm shows higher costs and greater effectiveness than the base case scenario (Figure 3).

The acceptability curve indicates that from a willingness to pay USD 663.06 to avoid a false negative test, 50% of simulations would favor the new diagnostic strategy than SSS microscopy by itself. This percentage would rise to 90% in simulations from USD 1,313 upward (Supplementary Material - Figure S6; https://cadernos.ensp.fiocruz.br/static//arquivo/suppl-e00038723_8950.pdf).

## Discussion

Leprosy remains a global public health problem despite the global efforts to eradicate it. Brazil endures a significant burden due to it and has not yet to meet WHO control goals. Early diagnosis configures a fundamental strategy to ensure the interruption of its transmission and prevent its disabling effects ^34^. However, no gold standard to diagnose the disease has been found since clinical examination investigates skin lesions with sensitivity changes, peripheral nerve involvement, sensory and motor changes, and slit-skin smear microscopy ^11^.

The diagnosis of leprosy based solely on SSS microscopy requires experienced professionals who can recognize the disease and its signs (which, in many cases, resemble those of other dermatological diseases). Moreover, the lack of a specialized infrastructure and human resources in the areas most affected by the disease hinder case detection and reporting ^35^. Referral to specialized centers can be time-consuming and requires the transport of patients, which can be complex, especially in remote areas. A Brazilian study ^36^ that included 116 patients found that about 10% of them waited from two to six months since testing for a confirmed leprosy diagnosis. Delayed diagnoses negatively affect prognosis and increase the risk of transmission, especially among household contacts ^37^.

This context entails crucial improvements to diagnostic capacity at primary care by developing rapid leprosy serological testing to meet this need by providing a simple and quick way to identify possible cases, increasing the diagnostic capacity of primary care, and enhancing the population’s access to health care. The RT and PCR tests have emerged as complementary strategies to diagnose leprosy ^38^. Although the accuracy of the serological test remains under debate, its use with clinical examinations and SSS microscopy following a diagnostic flow could aid the early detection of leprosy cases, especially among close contacts ^39^.

PCR has high sensitivity and specificity to detect *M. leprae* infections ^39^, which is very useful for PB cases ^40^. Although the isolated use of PCR could be cost-effective (Table 2), its large-scale employment would be unfeasible, especially in more isolated regions as it requires a laboratory infrastructure and trained professionals ^41^. Moreover, PCR remains unvalidated for use in skin scrapings and requires a biopsy, making exams more complex. The hypothetical scenario in which all suspects are tested with SSS microscopy followed by PCR in negative cases would provide the most cost-effective strategy if included in the model. However, it was decided to ignore this strategy due to its infeasibility in Brazil. SSS is painful and scarcely available in primary care. Thus, it would be unreasonable to expect that all patients would undergo this test as it would limit the quick reduction of the number of underdiagnosed patients with leprosy.

The RT, on the other hand, can be used even before the onset of the first lesions. It has a lower cost than PCR, is easy to perform, produces results immediately, and dispenses with special equipment or refrigeration. Moreover, it can classify patients as PB or MB, which is very useful for designing therapeutic interventions ^17^. However, its sensitivity and specificity vary widely ^35^. Its sensitivity is especially low in PB patients due to their low bacterial load, and only a low percentage of patients who have anti-PGL-I antibodies develop the disease. The isolated use of this test should be avoided as it would increase the number of false positives and unnecessary treatments and contribute toward antibacterial resistance ^40^. However, the high negative predictive value of anti-PGL-I serological tests for leprosy implies that negative tests will unlikely occur in MB patients and transmitters, making it a useful tool for the initial screening of close contacts ^38^.

Despite the scarce literature on clinical outcomes from complementary diagnostic tests in leprosy, it is reasonable to suppose that greater diagnostic capacity would contribute to reduce transmission. No diagnostic test, by itself, can diagnose leprosy as test results suffer the influence of the prevalence and clinical form of the disease ^23^ ^,^ ^40^. However, its combined use may represent an important advance in the diagnosis of the disease. The results of our economic analysis corroborate the decision by the Brazilian Ministry of Health to recommend a new diagnostic algorithm as it suggests the combination of rapid serological, SSS microscopy, and PCR tests as more cost-effective than the diagnostic procedure routinely used in Brazil (SSS microscopy by itself). The sequential use of these tests reduces the number of false-negative diagnoses at a relatively low cost.

The model this study proposed has some limitations. The available literature on the sensitivity and specificity of diagnostic tests is heterogeneous as studies have evaluated the accuracy of several types of RT and PCR techniques with different primers. Moreover, these studies were conducted in populations with different epidemiological profiles. This variability hindered the definition of the parameters to be used in our model and contributed to the degree of uncertainty in our results. Moreover, evidence on the incidence and prevalence of leprosy in the target population (intradomiciliary contacts of confirmed cases) is absent in the literature and unavailable from the Brazilian Ministry of Health. Thus, this study obtained these parameters from an expert panel. Although this source of evidence has limitations, it is worth noting that experts from the Brazilian Ministry of Health took part in the panels that derived the necessary parameters for this analysis. Even though the use of a correction factor for the cost of the SSS microscopy could also be considered a limitation, it lies beyond the scope of this study to discuss the methods to determine this factor or its external validity. This adjustment was made based on Brazilian Ministry of Health recommendations, which establishes that cost parameters from SIGTAP should undergo a 2.8-factor correction. Nevertheless, univariate sensibility analysis showed that this factor had an irrelevant impact on ICER. Finally, the analysis can be considered conservative because calculations ignored the benefits and costs of reducing sequelae by early diagnoses.

Despite its limitations, this study obtained important results. Leprosy is an endemic and neglected disease and the literature still has little information of its impact either on patients’ quality of life or in economic terms despite the efforts of international organizations and governments of affected countries. The burden of leprosy in Brazil remains significant and the country has not yet to eradicate it. The new diagnostic flow by the Brazilian government is a major step to enhance the early diagnosis of the disease and avoid transmission. The *Brazilian National Guidelines for Leprosy* ^6^ already recommend it and the Brazilian Ministry of Health centralizes the financing and acquisition of RT and PCR kits. Tests are distributed to states and municipalities according to an annual plan following epidemiological and consumption data. Therefore, assessing the benefits and costs of this intervention is relevant. To our knowledge, this is the first economic model to analyze the diagnostic flow of leprosy. We hope it will assist the Brazilian government to prioritize resource allocation based on economic evidence, favoring the feasibility and efficiency of its health system.
